# Unusual Suspect Causing Cavitary Pneumonia in a Vape Smoker

**DOI:** 10.7759/cureus.102582

**Published:** 2026-01-29

**Authors:** Mohamed M Darwish, Afnan Chaudhry, Mohammad Alazzeh, Obaid Rehman, Roopika Reddy

**Affiliations:** 1 Internal Medicine, Tower Health Medical Group, Phoenixville, USA; 2 Pulmonology and Critical Care, Tower Health Medical Group, Phoenixville, USA

**Keywords:** actinomyces infections, actinomyces odontolyticus, cavitary lung lesion, e-cigarettes, electronic cigarettes, e-smoking, smoking versus vaping, vaping

## Abstract

*Actinomyces* species are facultative anaerobic bacteria that are part of the normal flora of the human oral cavity, gastrointestinal tract, and vaginal tract. These bacteria are known to cause actinomycosis, a rare chronic granulomatous infection that typically presents as a slowly progressive disease. Pulmonary actinomycosis, although rare, has long been recognized as a distinct form of pneumonia, typically affecting individuals with underlying risk factors such as poor oral hygiene, aspiration risk, pre-existing lung disease, or immunocompromising conditions. The recent surge in electronic cigarette (e-cigarette) use has prompted concerns regarding its potential role in predisposing individuals to pulmonary infections, including those caused by commensal oral flora such as *Actinomyces* species. Here, we present a case of a 48-year-old immunocompetent woman with no significant medical conditions or risk factors aside from a five-year history of vaping, who developed a cavitary pneumonia caused by *Actinomyces odontolyticus*.

## Introduction

*Actinomyces* species, specifically *Actinomyces odontolyticus*, are facultative anaerobic bacteria that are part of the normal flora of the human oral cavity, gastrointestinal tract, and vagina. These bacteria are known to cause actinomycosis, a rare chronic granulomatous infection that typically presents as a slowly progressive disease. Actinomycosis most commonly affects immunocompromised individuals, and is categorized by the anatomical site of involvement, with cervicofacial (60%), abdominal (20%), pulmonary (15%), and pelvic (5%) forms being most prevalent [1].

Pulmonary actinomycosis, although rare, has long been recognized as a distinct form of pneumonia, typically affecting individuals with underlying risk factors such as poor oral hygiene, aspiration risk, pre-existing lung disease, or immunocompromising conditions [2]. The recent surge in electronic cigarette (e-cigarette) use has prompted concerns regarding its potential role in predisposing individuals to pulmonary infections [3]. Here, we highlight vaping as a risk factor in the development of atypical lung infections. We present a case of a 48-year-old immunocompetent woman with no significant medical conditions or risk factors, aside from a five-year history of vaping, who developed a cavitary pneumonia caused by *Actinomyces odontolyticus* and was successfully treated with oral penicillin V. The patient has provided consent to report this case.

## Case presentation

A 48-year-old obese woman with a past medical history of opioid use on methadone therapy and a five-year history of vaping presented to the emergency department with a one-week history of dry cough and shortness of breath. She denied fever, chills, night sweats, hemoptysis, or weight loss, and she had no recent upper respiratory symptoms such as rhinorrhea, sore throat, or nasal congestion. On examination, the patient was afebrile and did not exhibit signs of respiratory distress but had oxygen saturation of 87% on room air. The physical examination of the chest had no significant findings.

**Figure 1:**
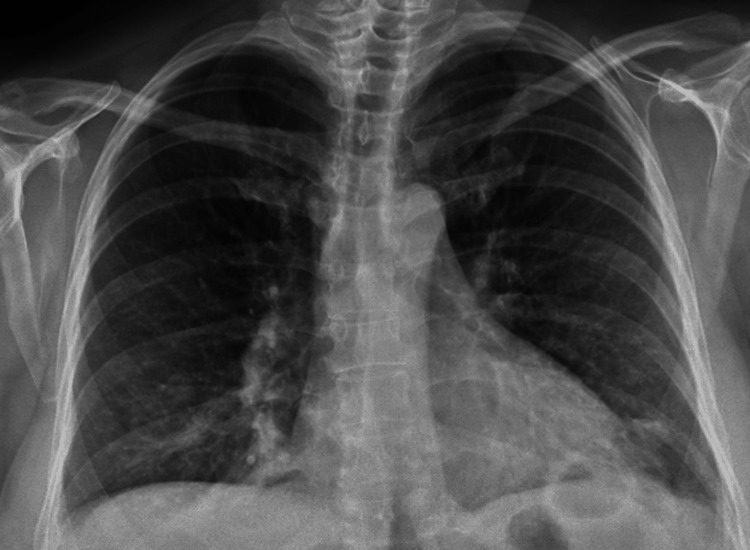
Chest X-ray on presentation, reported to have no acute lung pathology

**Figure 2:**
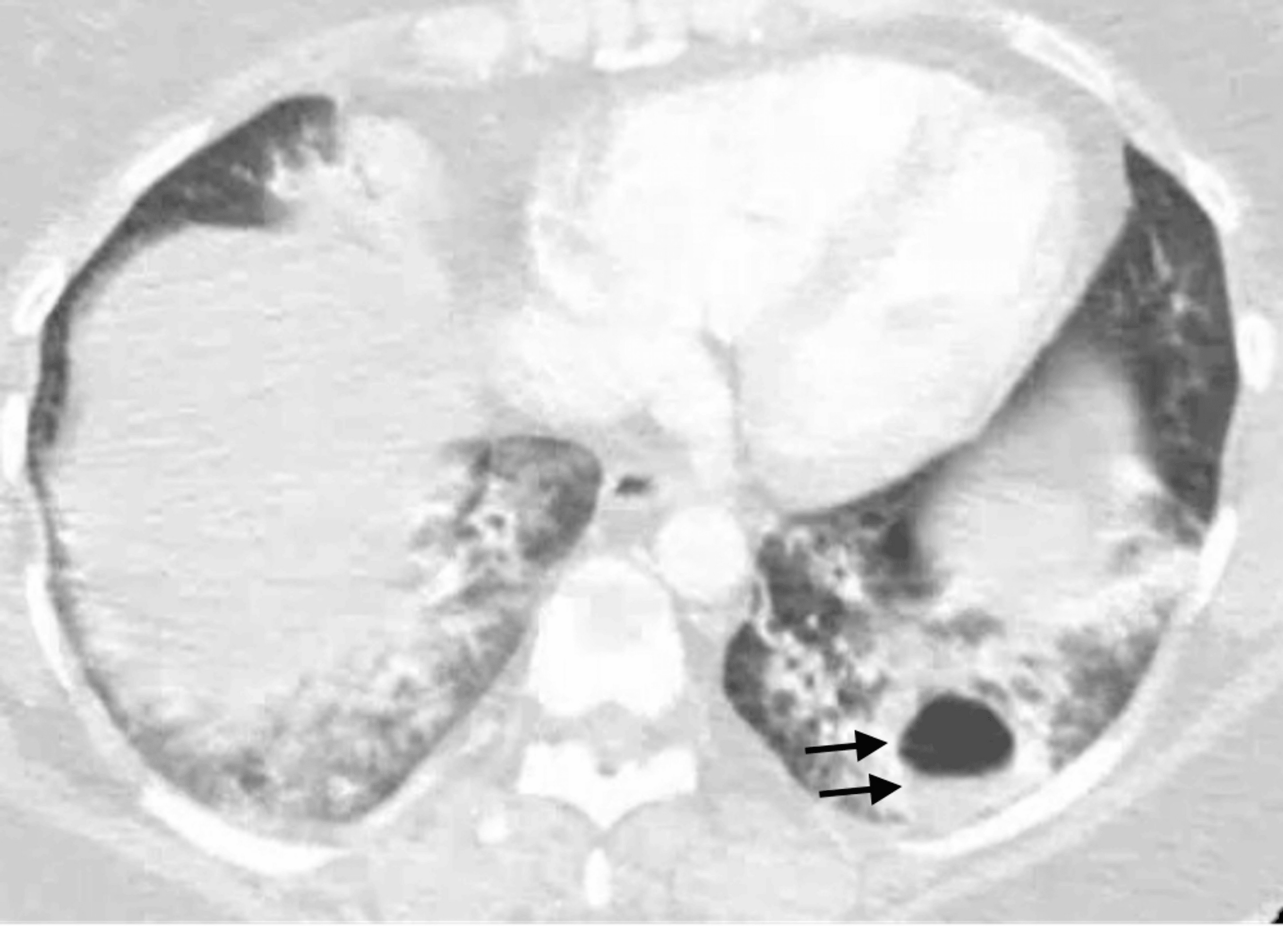
Chest CT showing left lower lobe cavitary lesion with air-fluid level and bilateral ground glass opacities

Chest radiograph (Figure 1) was performed, revealing bibasilar opacities and prompting further investigation with a CT angiography of the chest (Figure 2), which showed patchy ground glass opacification in both lower lobes with a left lower lobe cavitary lesion with air-fluid level suggestive of an abscess, fungal infection, cyst, or tuberculosis.

Given the severity of the imaging findings, she was started on broad-spectrum antibiotics. The following day, a bronchoscopy with bronchoalveolar lavage (BAL) was performed, along with a brush sample from the posterior segment of the left lower lobe. Cultures of the BAL fluid grew *Actinomyces odontolyticus* in pure culture.

Upon further history-taking based on the findings, the patient stated that she had recently undergone a root canal procedure, although she denied any associated dental pain or discharge. A panorex dental X-ray was performed, which showed no obvious signs of infection at the site of the root canal treatment. Treatment with oral penicillin V 2 g was initiated for three months period. The patient's symptoms were improving until discharge, and the patient was scheduled for a follow-up appointment, which confirmed the clearance of the infection.

## Discussion

Traditionally, actinomycosis is associated with chronic conditions that compromise immune function, such as diabetes mellitus, alcoholism, and poor oral hygiene. However, as evidenced by this case, pulmonary actinomycosis can occur in healthy, immunocompetent individuals with the presence of risk factors such as root canal procedure and vaping, which will facilitate the entry of *Actinomyces odontolyticus* into the lower respiratory tract [2].

Vaping, a relatively recent phenomenon, has raised significant concerns about its potential long-term effects on respiratory health. Nicotine and other chemical additives in e-cigarettes have been shown to alter the normal respiratory microbiome, impair mucociliary clearance, and induce inflammatory responses that impair the lung's defense mechanisms [4]. Vaping has been shown to elevate oxidative stress in lung cells, which may impair immune responses and enhance bacterial survival in the lungs, making the act of "inhalation" a potential vector that facilitates the entry of oral pathogens into deeper respiratory structures. Another presumed pathophysiological mechanism that distinguishes vaping from smoking is the ability to cause micro-aspirations due to the high inhalation force in vaping. A recent study highlighted a case in which vaping marijuana was linked to pulmonary actinomycosis, which is found in the oral cavity. This case adds to the growing body of evidence that vaping predisposes healthy individuals to atypical lung infections [5].

The clinical presentation of pulmonary actinomycosis is often nonspecific and can include persistent cough, hemoptysis, shortness of breath, fever, or vague insidious symptoms, which can delay the diagnosis. Imaging findings such as cavitary lesions, pleural effusions, and consolidation can mimic other conditions such as tuberculosis, fungal infections, and malignancies [6]. While bronchoalveolar lavage (BAL) aided in the diagnosis by culturing and growing the organism, the diagnostic challenge arises in the slow-growing nature of *Actinomyces*, making culture isolation difficult and time-consuming [7]. In fact, studies show that diagnosis is often delayed, with a correct diagnosis made in only 4% of cases at the initial presentation [8]. Therefore, in such cases of atypical pneumonia with a cavitary lesion, inquiring about risk factors such as vaping and dental manipulation can direct clinicians to consider commensal flora such as actinomycosis as a causative organism. Furthermore, clinicians should be aware of the emerging risks posed by vaping in patients with respiratory symptoms and consider it as a potential contributing factor when evaluating patients.

## Conclusions

This case underscores the importance of considering vaping as a risk factor predisposing to lower respiratory infections especially by organisms that are otherwise nonharmful and inhabiting the upper respiratory tract. These organisms might translocate into the lungs via multiple mechanisms, including the act of inhalation. Continued research is warranted into the effects of vaping as a risk factor to predispose individuals to lung infections by commensal bacterial flora.

## References

[1] Zhang M, Zhang XY, Chen YB (2017). Primary pulmonary actinomycosis: a retrospective analysis of 145 cases in mainland China. Int J Tuberc Lung Dis.

[2] Valour F, Sénéchal A, Dupieux C (2014). Actinomycosis: etiology, clinical features, diagnosis, treatment, and management. Infect Drug Resist.

[3] Corriden R, Moshensky A, Bojanowski CM, Meier A, Chien J, Nelson RK, Crotty Alexander LE (2020). E-cigarette use increases susceptibility to bacterial infection by impairment of human neutrophil chemotaxis, phagocytosis, and NET formation. Am J Physiol Cell Physiol.

[4] Esteban-Lopez M, Perry MD, Garbinski LD (2022). Health effects and known pathology associated with the use of e-cigarettes. Toxicol Rep.

[5] Massey M, Barney J (2021). Pulmonary actinomycosis and marijuana vaping. BMJ Case Rep.

[6] McKee N, Carson MI, Ross C (2024). The diagnosis and management of pulmonary actinomycosis in an immunocompetent patient. Chest.

[7] Boyanova L, Kolarov R, Mateva L, Markovska R, Mitov I (2015). Actinomycosis: a frequently forgotten disease. Future Microbiol.

[8] Wong VK, Turmezei TD, Weston VC (2011). Actinomycosis. BMJ.

